# Studies on Viroid Shed Light on the Role of RNA Three-Dimensional Structural Motifs in RNA Trafficking in Plants

**DOI:** 10.3389/fpls.2022.836267

**Published:** 2022-03-23

**Authors:** Junfei Ma, Ying Wang

**Affiliations:** Department of Biological Sciences, Mississippi State University, Starkville, MS, United States

**Keywords:** viroid, RNA motif, RNA nuclear import, RNA cell-to-cell trafficking, RNA systemic trafficking

## Abstract

RNAs play essential roles in various biological processes. Mounting evidence has demonstrated that RNA subcellular localization and intercellular/systemic trafficking govern their functions in coordinating plant growth at the organismal level. While numerous types of RNAs (i.e., mRNAs, small RNAs, rRNAs, tRNAs, and long noncoding RNAs) have been found to traffic in a non-cell-autonomous fashion within plants, the underlying regulatory mechanism remains unclear. Viroids are single-stranded circular noncoding RNAs, which entirely rely on their RNA motifs to exploit cellular machinery for organelle entry and exit, cell-to-cell movement through plasmodesmata, and systemic trafficking. Viroids represent an excellent model to dissect the role of RNA three-dimensional (3D) structural motifs in regulating RNA movement. Nearly two decades of studies have found multiple RNA 3D motifs responsible for viroid nuclear import as well as trafficking across diverse cellular boundaries in plants. These RNA 3D motifs function as “keys” to unlock cellular and subcellular barriers and guide RNA movement within a cell or between cells. Here, we summarize the key findings along this line of research with implications for future studies on RNA trafficking in plants.

## Introduction

Multicellular organisms evolve diverse mechanisms to integrate individual cells during development and in response to environmental cues. Cellular boundaries function in this integration through balancing cell autonomy and communication among cells (Ding and Wang, 2009). In plants, neighboring cells are connected *via* plasmodesmata (PD), which are micro-channels crossing cell walls. The vascular system, including xylem and phloem, mediates the systemic transportation of molecules. The xylem system is mainly responsible for the transportation of water and minerals, while the phloem system transports photosynthates and macromolecules. Various proteins, RNAs, as well as viruses and viroids can be found in the translocation stream of phloem (Ding et al., 2003; Liu and Chen, 2018).

Most cellular RNAs are transcribed in the nucleus and then are either retained in the nucleus or transported to the cytoplasm for function. RNAs in the cytoplasm can participate in diverse biological activities and processes or are transported to the nucleus performing various functions, or even traffic to neighboring cells to act as non-cell-autonomous regulators (Ye et al., 2012; Axtell, 2013; Chantarachot and Bailey-Serres, 2018; Reagan et al., 2018; Long et al., 2021). Non-cell autonomous RNAs widely exist in plants, and there are many types of those trafficking RNAs, including various small RNAs, mRNAs, tRNAs, rRNAs, and infectious RNAs from viruses and viroids (Ding et al., 2003; Liu and Chen, 2018). Some mobile RNAs will move short distance across several cells, while others move through various tissues to traffic systemically in plants (Kim, 2005; Qin et al., 2012; Spiegelman et al., 2013; Thieme et al., 2015; Yang et al., 2015; Ham and Lucas, 2017; Reagan et al., 2018).

Non-cell autonomous RNAs serve as critical signals to regulate plant development and responses to biotic and abiotic challenges (Ding et al., 2003; Wang and Ding, 2010; Liu and Chen, 2018). Using grafting experiments, some mRNAs are found to traffic long distance across the grafting junctions in regulating plant development, such as tuber formation in potato (Banerjee et al., 2006) and leaf morphogenesis in tomato (Kim et al., 2001; Haywood et al., 2005). Related to plant physiology, a microRNA (miR399) has been found to move from shoot to root contributing to the maintenance of phosphate homeostasis in *Arabidopsis* (Lin et al., 2008; Pant et al., 2008). Numerous small RNAs, including miRNAs and short interfering RNAs (siRNAs), serve as long distance epigenetic signals coordinating gene expression and antiviral defense (Ding and Voinnet, 2007; Kalantidis et al., 2008; Chen and Rechavi, 2021).

A key question remains regarding how RNA is selected for trafficking (including intracellular, intercellular, and systemic trafficking). A recent report showed that m^5^C methylation is highly enriched in mobile mRNAs. Loss-of-methylation inhibits the non-cell-autonomous behavior of some mobile mRNAs (Yang et al., 2019). This finding provides mechanistic insights into the selection specificity of mobile transcripts. However, it is unclear whether m^5^C methylation ensures the accurate transportation of RNAs to their proper destiny within plants. RNAs by themselves also contain signals in regulating long distance RNA trafficking. For example, a *cis* element cloned from the 5′ untranslated region (UTR) of a potexviral RNA mediates cell-to-cell movement of the fused GFP reporter RNA (Lough et al., 2006). In addition, the UTRs of potato *BEL5* mRNA possess the regulatory elements for long distance trafficking (Banerjee et al., 2006). The detailed molecular basis underlying these functional structures remains to be determined.

## Viroids as a Productive Model to Understand Structure-Based RNA Trafficking

Viroids, single-stranded circular noncoding RNAs, harness cellular machinery to target specific organelles for replication, invade neighboring cells through PD, and spread systemically *via* phloem (Ding, 2009). Within phloem, viroids likely form an RNA-protein complex with phloem pectin PP2 for long distance translocation (Gómez and Pallás, 2001; Owens et al., 2001; Gomez and Pallas, 2004). During systemic infection, viroid RNAs move across various cellular boundaries (Ding and Wang, 2009; Wang and Ding, 2010). In a simplified view, viroids will traffic from epidermis, through palisade and spongy mesophyll and bundle sheath, to enter phloem. They also traffic in a reverse direction in systemic leaves (Takeda and Ding, 2009). Viroids accumulate to high levels in plant cells, and it is easy to engineer various mutants for functional analyses (Wang and Ding, 2010). There is no endogenous background signal interfering with analyses on viroid RNAs. Therefore, viroid infection provides a valuable experimental system to dissect the factors and regulatory mechanisms underlying RNA movement in plants.

Given that viroids do not encode any proteins, their RNA genomes must contain explicit information to guide cellular machinery for accurate localization and trafficking. Using potato spindle tuber viroid (PSTVd) as a model, specific RNA three-dimensional (3D) motifs responsible for crossing multiple cellular boundaries have been identified, providing solid genetic evidence that those RNA motifs guide specific trafficking in plants (Ding, 2009; Wang et al., 2018, 2021). It is noteworthy that the secondary structure of viroid RNAs is among the best-known structures thanks to the extensive chemical mapping analyses (Gast et al., 1996; Xu et al., 2012; Giguere et al., 2014; Giguere and Perreault, 2017; Lopez-Carrasco and Flores, 2017), which paves the way to further pinpoint the functional structures of local RNA 3D motifs.

## Essential Role of Non-Watson-Crick Base-Pairing in RNA Loop Motifs

RNA molecules form various helices and loops in their secondary structures. Helices are composed of contiguous Watson-Crick (WC) base pairs [i.e., adenine (A)–uridine (U), guanine (G)–cytosine (C), and GU base pairs]. In contrast, loop regions are composed of diverse non-WC base pairs that are highly arranged (Wang et al., 2018). Many loop motifs can be found at nonhomologous positions of diverse RNA species but largely keep the base pair geometries and interaction details, so they are also termed recurring loop motifs (Khisamutdinov et al., 2021). RNA 3D loop motifs provide recognition sites for specific RNA-protein, RNA–RNA, and RNA-ligand interactions. This is feasible thanks to the non-WC-WC base pairs that widen the major groove of RNAs and expose distinct WC edges of four bases (Wang et al., 2018).

**Figure 1:**
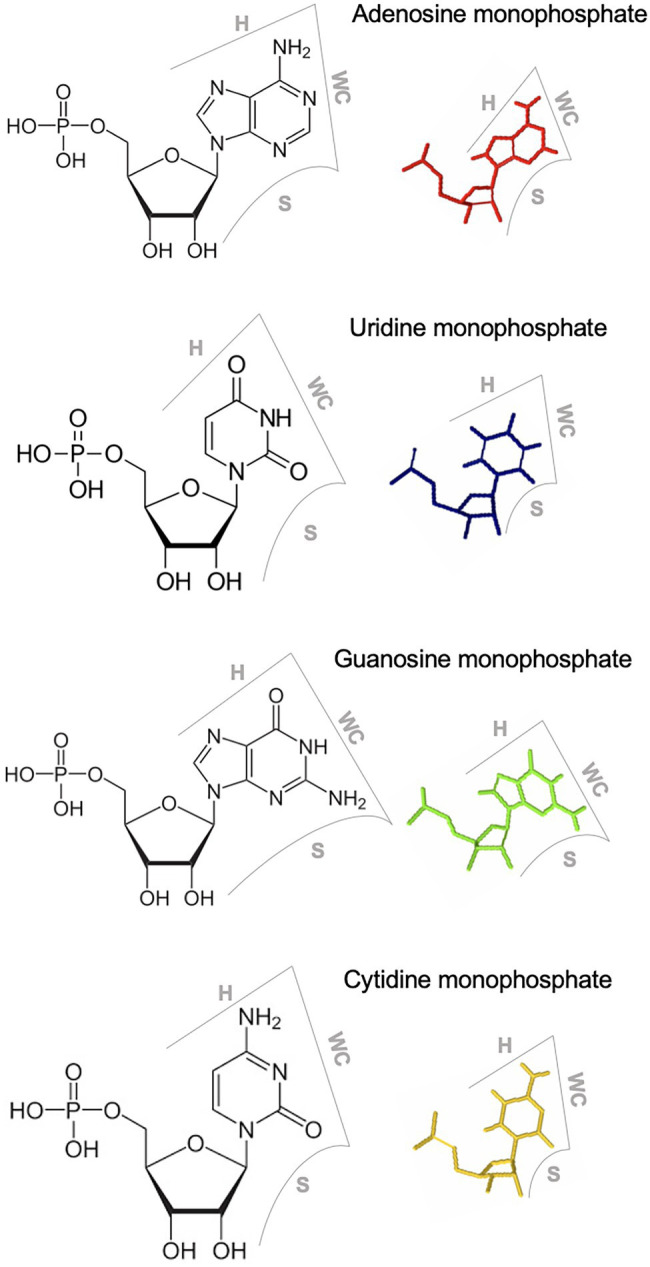
Three edges of RNA nucleotides. WC, Watson-Crick edge; H, Hoogsteen edge; S, sugar edge.

Each RNA base has three edges (i.e., the WC, Hoogsteen, and Sugar edges) that can participate in interaction with other base edges to form non-WC-WC base pairs in loop motifs (Figure 1). Adding to the complexity are the two possible orientations (*cis* as the same orientation and *trans* as the opposite orientation) of glycosidic bonds for each base pair. Therefore, there are 12 base-pairing geometries considering the combinations of interacting edges and glycosidic bond orientations (Stombaugh et al., 2009; Wang et al., 2018). Two base pairs are isosteric to each other if they are formed *via* the same base edge interactions, share the same glycosidic bond orientations, and possess highly similar distances between C1’ carbon atoms (Stombaugh et al., 2009; Wang et al., 2018). Isosteric base pairs have the potential to substitute for each other without disrupting functions. These geometries and isostericity in various loop motifs have been empirically observed in detail through atomic-resolution crystallography and NMR spectroscopy studies (Stombaugh et al., 2009; Wang et al., 2018). Efforts have been made to summarize those observed geometries, which are deposited in the RNA Basepair Catalog (Stombaugh et al., 2009). In addition, some prediction programs have been developed to facilitate the annotation of RNA loop motifs in terms of base geometries and interactions therein (Sarver et al., 2008; Zirbel et al., 2015). Therefore, it is possible to deduce the base interaction geometry within RNA loops and design structure-disruptive or structure-maintaining mutants for functional validations of predicted structural models of RNA loop motifs, providing genetic evidence in supporting some analyzed RNA 3D motifs.

## A C-Loop for RNA Nuclear Import

Viroids of the family *Pospiviroidae* all enter the nucleus for replication. How RNA nuclear import is regulated remains as an interesting question. Most RNAs are made in the nucleus, and the prevailing view is that those RNAs either traffic to the cytoplasm or remain in the nucleus for function. In recent years, more and more RNAs, besides viroids, have been found to enter the nucleus (Rudt and Pieler, 1996; Chou et al., 1998; Gao et al., 2012; Ye et al., 2012; Kramer and Hopper, 2013; Chaturvedi et al., 2014; Long et al., 2021). Using PSTVd as a model, we found that viroids exploit cellular importin alpha-4 (IMPa-4)-based pathway and a viroid-binding protein (Virp1) to achieve nuclear import (Ma et al., 2021).

**Figure 2:**
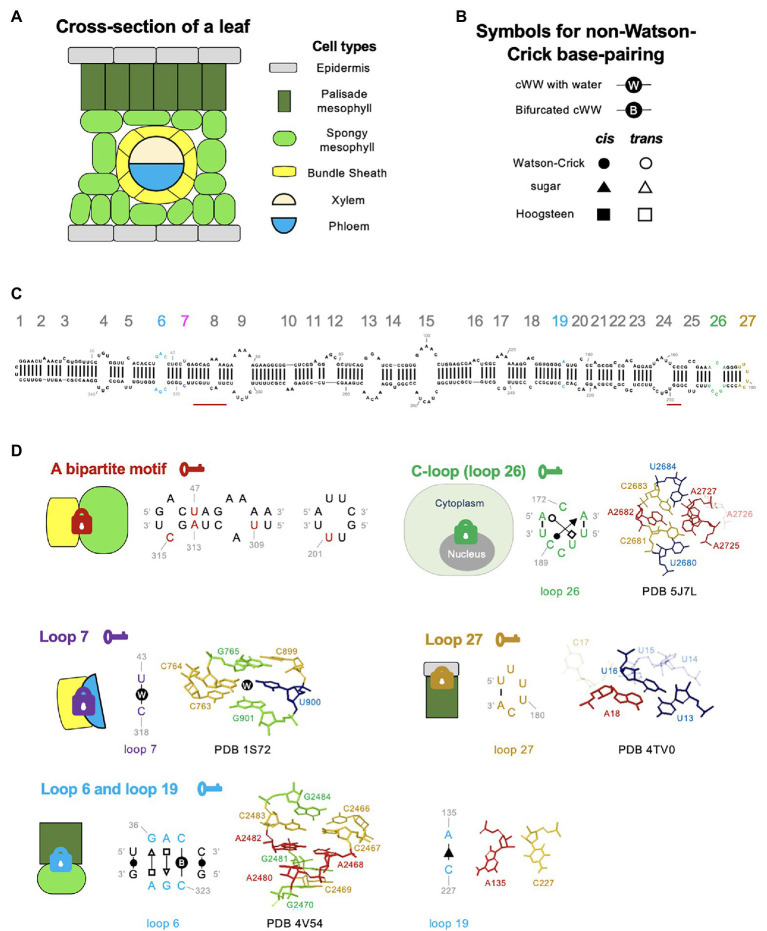
RNA 3D motif-mediated viroid RNA trafficking in plants. **(A)** A cartoon illustration of the cross-section of plant leaves. **(B)** Symbols for annotating RNA nucleotide edges. cWW, cis-Watson Crick-Watson Crick base-pairing. **(C)** The secondary structure of the PSTVd^Int^ genome, drawing by using RNA2Drawer (Johnson et al., 2019). Loops and bulges are numbered from left to right as 1 to 27. Key structural motifs illustrated in **(D)** are highlighted in colors (nucleotides and loop numbers). The nucleotides of the bipartite motif (position indicated by two red bars) are not color-highlighted in the secondary structure because this motif was only found in the PSTVd^NB^ strain, whose secondary structure has not been confirmed by chemical probing. **(D)** Loop structures critical for regulating trafficking across cellular boundaries. Locks with different colors indicate distinct barriers that restrain the trafficking of RNAs without necessary RNA motifs (“keys”). The annotated base-pairings were validated by chemical mapping and functional mutagenesis. The C-loop structure model is based on regions 2,680–2,684 (5′-UCACU-3′) and 2,725–2,727 (5′-AAA-3′) of bacterial 23S rRNA (PDB 5J7L; Cocozaki et al., 2016). The loop 7 structural model is based on regions 763–765 (5′-CCG-3′) and 899–901 (5′-CUG-3′) of *Haloarcula marismortui* 23S rRNA (PDB 1S72; Klein et al., 2004). The loop 27 model is based on the region 13–18 (5′-UUUUCA-3′) of a Drosophila histone mRNA (PDB 4TV0; Zhang et al., 2014). The loop 6 model is based on regions 2,466–2,470 (5′-CCACG-3′) and 2,480–2,484 (5′-AGACG-3′) of bacterial 23S rRNA (PDB 4V54; Borovinskaya et al., 2007). Note that C2483 and C2467 form a bifurcated cWW base-pairing. The illustration for loop 19 is extracted from the RNA Basepair Catalog. Nucleotides in transparent indicate that they are not involved in base interactions (e.g., A2726 in PDB 5J7L).

Previous studies have mapped a region in viroid RNA genomes responsible for Virp1 binding, termed RY motif (Gozmanova et al., 2003; Maniataki et al., 2003). However, the molecular basis of the RY motif for Virp1 recognition remains elusive. Recently, we carefully re-examine the RY motif in PSTVd and uncovered a C-loop structure within the region. C-loop is an asymmetric loop that has been found in many rRNAs, mammalian noncoding RNAs and one bacterial mRNA (Torres-Larios et al., 2002; Lescoute et al., 2005; Iacoangeli and Tiedge, 2013; Drsata et al., 2017). C-loop has the following features: (A) “C” is often the first base in the longer strand; (B) two bases in the longer strand form non-WC base pairs with bases in the opposite strand (*cis*-WC-sugar and *trans*-WC-Hoogsteen base-pairings); (C) two triads are formed through base-pairings from two strands; and (D) this motif is often found in hairpin stem-loop structure (Lescoute et al., 2005; Drsata et al., 2017). PSTVd C-loop is composed of C189-A173 *cis*-WC-sugar base-pairing and A171-U187 *trans*-WC-Hoogsteen base-pairing (Figure 2), which is supported by chemical mapping data and functional mutagenesis analyses (Ma et al., 2021). Our data showed that C-loop is pivotal for Virp1-binding, viroid nuclear accumulation, and infectivity. Interestingly, C-loop can be found in nearly all, except one, formal members of the family *Pospiviroidae* as well as a viral satellite RNA that relies on Virp1 for nuclear import (Ma et al., 2021). Therefore, C-loop is probably a widely used RNA motif for the nuclear import of subviral RNAs. Given that the nuclear import of RNAs is not limited to subviral agents, this finding will encourage new efforts to cast a wider net in search for more regulatory RNA motifs responsible for nuclear import.

## A Bipartite Structure Mediating the Exit of Bundle Sheath

It is intuitive to reason that viroid RNAs contain the necessary information for cell-to-cell and even long distance trafficking. Early work using PSTVd sequence in chimeric RNAs supports this hypothesis (Ding et al., 1997). Analyses on PSTVd^NT^ and PSTVd^NB^ strains provided the first empirical evidence illustrating an RNA motif responsible for regulating trafficking across cellular boundaries (Qi et al., 2004). PSTVd^NT^ harbors a spontaneous nucleotide substitution C259U in the tomato isolate PSTVd^KF440-2^ that enables infection in tobacco (*Nicotiana tabacum*; Wassenegger et al., 1996). PSTVd^NB^ accumulated five more spontaneous mutations (A47U, G210U, A309U, U313A, and U315C) during vegetative propagation through cuttings from PSTVd^NT^-infected tobacco plants (Qi et al., 2004). PSTVd^NT^ and PSTVd^NB^ bear similar replication efficiency in protoplasts but display different accumulation levels in systemic leaves. *In situ* hybridization analyses showed that PSTVd^NB^, but not PSTVd^NT^, can exit the bundle sheath to invade more cells in systemic leaves (Qi et al., 2004).

Mutational analyses found that four out of the five spontaneous mutations in PSTVd^NB^ are both required and sufficient to enable bundle sheath exit in tobacco. Interestingly, these substitutions are clustered in two discrete regions in the PSTVd genome forming a bipartite motif (Figure 2; Qi et al., 2004). The detailed structural basis of this bipartite motif remains elusive. It is noteworthy that this bipartite motif appears to be required for unidirectional bundle sheath exit only in tobacco but not in tomato or *N. benthamiana*, indicating that those RNA motifs can evolve very fast in nature and influence the host tropism of infectious RNAs.

## An RNA Motif for Phloem Loading

An early attempt to screen for PSTVd loss-of-function trafficking mutants identified two nucleotides (U43 and C318) that did not affect replication but abolished systemic trafficking (Zhong et al., 2007). Interestingly, U43 and C318 form a one base-pairing loop, loop 7 (Figure 2). Using *in situ* hybridization analyses found that loop “close” mutants (U43G or C318A) were present in epidermis, mesophyll, and bundle sheath cells but could not be loaded into phloem in inoculated leaves. It is unclear whether this regulation is unidirectional or bidirectional because transgenic expression of loop 7 mutants in companion cells all converted to wild-type sequences, rendering it difficult to assess the regulation of trafficking direction.

Using the FR3D program to search for similar loop structures obtained by highest-resolution X-ray crystallography in the Protein Data Bank (PDB) revealed that U43 and C318 form a *cis*-WC–WC base pair with a water molecule insertion (Figure 2; Zhong et al., 2007). All the mutants predicted to retain the structure for water insertion were able to traffic systemically in plants, whereas the mutants predicted to form canonical WC–WC base-pairing without water insertion failed to traffic out of inoculated leaves. The water molecule insertion widens the minor groove and increases the angle subtended by the glycosidic bonds, presumably favoring protein binding (Zhong et al., 2007). Indeed, similar structures in rRNAs and siRNA duplexes are involved in protein binding (Vargason et al., 2003; Klein et al., 2004).

## Genome-Wide Analyses Uncovering Multiple Loops in PSTVd Regulating Systemic Trafficking

The discovery of loop 7 and the bipartite motif inspired the idea that there exist elegant regulations at each cellular boundary regulating the exchange of cellular contents, including RNAs. Accordingly, multiple RNA motifs will work in concert to coordinate trafficking across those cellular boundaries. Therefore, genome-wide functional analyses of PSTVd loop motifs were performed to assess the role of each loop motif in regulating replication or systemic trafficking (Zhong et al., 2008). By replacing all possible noncanonical base-pairings in loop motifs with WC–WC base-pairings, a series of mutants were generated to “close” every loop motif except loop 15 and loop 7 that have been previously analyzed. These loop mutants were tested for replication ability in protoplasts and systemic trafficking in *N. benthamiana* plants. A total of 11 loop mutants were found to impair systemic trafficking (Zhong et al., 2008). Some of the loops, such as loop 6, loop 19, and loop 27, were subsequently found to regulate the trafficking across distinct cellular boundaries (see below for details). It is noteworthy that this analysis may overlook some more complex structures, such as the aforementioned bipartite motif. In addition, loop 26 mutant did not show trafficking because it regulates nuclear import as aforementioned (Ma et al., 2021). Nevertheless, this approach provides an overview of the genomic organization of viroids in controlling trafficking in *N. benthamiana*. Expanding this approach to other viroids, viruses, and diverse host-viroid combinations may achieve a much deeper understanding of structural motif-regulated RNA trafficking in plants.

## A UNCG-Like Motif Mediating Unidirectional Movement From Epidermis to Palisade Mesophyll

Upon inoculation, viroid inoculum will initiate replication and then move through mesophyll layers to enter phloem. A recent study showed that the right terminal loop (loop 27) of PSTVd is critical for RNA moving from epidermis to palisade mesophyll (Wu et al., 2019). Mutagenesis analyses showed that most mutants disrupting this loop led to failure in replication, except for the U178G/U179G mutation. The U178G/U179G mutant could spread within epidermis of inoculated leaves but was restrained from entering the adjacent palisade mesophyll layer as observed *via* the *in situ* hybridization assay. Interestingly, needle punch delivery of this mutant into stems allowed mutant RNA to move across all cellular boundaries including from palisade mesophyll to epidermis in systemic leaves, indicating that this loop regulates unidirectional trafficking (Wu et al., 2019).

Using the JAR3D program (Zirbel et al., 2015), the terminal loop is predicted to be a UNCG-like motif.^1^ The exact homolog loop can be found in the 3′ UTR of a Drosophila histone mRNA, where the loop is involved in protein binding (Zanier et al., 2002). Within the loop region (nucleotides U177 to A182), U179 and C181 bulge outside of the motif. U180 stacks on the WC–WC pair (U177-A182) that closes the motif (Figure 2). It is believed that this loop does not contain stable noncanonical base-pairings when the protein partner is absent (Wu et al., 2019).

## Two RNA Motifs for Movement Between Palisade and Spongy Mesophyll

When a similar *in situ* hybridization analysis was performed using loop 6 mutants to determine the role of loop 6 in regulating viroid movements, those mutants, if replicable, were trapped in palisade mesophyll cells without entering spongy mesophyll (Takeda et al., 2011). A more recent work on loop 19 mutants also revealed a similar pattern that loop 19 mutants accumulated in palisade mesophyll but could not enter spongy mesophyll (Jiang et al., 2017). It represents the first example where two RNA motifs regulate trafficking across the same cellular boundary. However, it cannot be ruled out that these two motifs form a larger bipartite organization to coordinate functions.

PSTVd loop 6 contains three noncanonical base pairs: C323-C38 *cis*-WC–WC bifurcated pair, G324-A37 *trans*-Sugar-Hoogsteen (tSH) pair, and A325-G36 *trans*-Hoogsteen-Sugar (tHS) pair (Figure 2; Takeda et al., 2011). The C323-C38 *cis*-WC–WC bifurcated pair is so rigid that it cannot be replaced by any other substitution. The tSH and tHS pairs can be substituted by some but not all isosteric base-pairings, implying the existence of other selection pressures (Takeda et al., 2011). Loop 6 is conserved in viroids belonging to the genus *Pospiviroid*. Similar loop motifs can also be found in some 16S rRNAs, 23S rRNAs, and a group I intron, where this structural motif serves as a binding site for protein partners (Takeda et al., 2011). Loop 19 is a one base-pairing loop (Takeda et al., 2018). This motif can emerge through spontaneous base substitutions in plants inoculated with loop-close mutants. Mutational analyses found that loop 19 is likely composed of a *cis*-Sugar-Sugar base-pairing (Figure 2; Takeda et al., 2018).

## Future Perspectives

Emerging evidence supports the model that multiple structural motifs coordinate RNA subcellular localization and trafficking across different cellular boundaries within a plant. Those structural motifs act like “keys” to unlock restrictions at organellar gates as well as various cellular boundaries. A reasonable hypothesis is that those RNA 3D motifs are recognized by certain cellular proteins forming RNA-protein complexes, which will then be delivered to their destinations. In support of this model, the PSTVd C-loop serves a nuclear import signal recognized by cellular protein Virp1. The Virp1-PSTVd complex is then delivered into the nucleus *via* the IMPa-4 based nuclear import pathway (Ma et al., 2021). In addition, recent evidence supports that an Exportin 5 ortholog (HASTY) participates in miRNA cell-to-cell and vascular movement in plants (Brioudes et al., 2021). On the other hand, the molecular basis of those barriers at various cellular boundaries remains to be determined. It is intuitive to reason that PD may adopt different selectivity when connect various types of cells. Diverse groups of proteins contribute to cargo targeting to PD and/or PD gating, including specific β-1,3-Glucanases for callose deposition, and other PD-associated or mobile proteins (Haywood et al., 2002; Ding et al., 2003; Levy et al., 2007; Brunkard et al., 2015; Lee, 2015). Those components may have different homologs or activities in distinct tissues, which can explain the need of multiple RNA motifs for crossing various cellular boundaries.

The complexity in organizing the required RNA motifs for RNA trafficking is intriguing. Some RNA motifs act in a species-specific manner while others work in concert to cross one specific cellular boundary, reflecting the sophisticated design in maintaining the autonomy of various tissues in different plants. To gain a deeper understanding of the barriers of cellular boundaries in different plants, analyzing the requirement of PSTVd trafficking motifs in different host-viroid combinations will be a straightforward approach to provide informative insights. Given the importance of RNA 3D motifs in host-viroid interactions, they certainly play a role in constraining viroid evolution and adaptation to new hosts (Wang et al., 2018). On the other hand, emerging evidence (e.g., bipartite as well as Loops 6 and 19) supports that viroid RNAs may undergo significant changes in overall structure to carry out functions. Currently, viroid RNA structures are mostly probed using *in vitro* assays. It will be beneficial to gain more insights into viroid structures at distinct subcellular and cellular compartments using *in vivo* probing methods, particularly those that can achieve observation at the single molecular level to detect the transient structural changes (Stephenson et al., 2016; Yu and Lucks, 2019; Cawte et al., 2020; Schmidt et al., 2020).

It is desirable that the knowledge gained from the viroid model can facilitate the understanding of cellular RNA trafficking. Increasing evidence supports that regulatory RNA structures can control endogenous RNA trafficking in plants, especially those tRNA-like structures (TLS) that have been identified in many endogenous mobile transcripts (Zhang et al., 2016). However, the detailed 3D base-pairing geometries, which confers the regulatory function in RNA trafficking in plants, have not been annotated for those regulatory structures. This is likely due to the technical limitations that hinders the in-depth analyses on such functional motifs. First, recurring RNA motifs may not exert the same function in different RNAs. For example, both PSTVd and 5S rRNA contain the loop E motif (Branch et al., 1985). However, a cellular protein, TFIIIA-9ZF, only binds the loop E in 5S rRNA (Szymanski et al., 2003) but prefers a distinct region instead of the loop E in PSTVd (Wang et al., 2016). Therefore, it is difficult to predict the function of recurring RNA motifs in distinct RNAs at this stage. Second, most cellular RNAs do not have well-annotated secondary structure, except for rRNAs, tRNAs, etc. Well-annotated secondary structures are a prerequisite for analyzing local 3D motifs. With the rapid development of novel probing methods for analyzing RNA structures at the transcriptome level (Gosai et al., 2015; Tack et al., 2020; Liu et al., 2021), this limitation may soon be mitigated.

The discovery of m^5^C methylation as a regulatory mark for mRNA translocation crossing graft junctions is a significant advancement in understanding the trafficking of endogenous RNAs (Yang et al., 2019). However, the m^5^C mark is enriched mostly four nucleotides downstream of the start codon in plant mRNAs (Song et al., 2018) and promotes the efficiency of mRNA translation (Tang et al., 2020). How the two biological processes (i.e., translation and selection for trafficking) are balanced remains to be elucidated. Viroid RNAs do not possess m^5^C modification (Di Serio et al., 2019), demonstrating that more than one mechanism exists for selecting mobile RNAs. How these mechanisms are coordinated for accurate delivery of RNAs to destiny remains unexplored, which deserves future investigations.

## Author Contributions

All authors listed have made a substantial, direct, and intellectual contribution to the work and approved it for publication.

## Funding

Work from the author’s laboratory was supported by research grants from the US National Science Foundation (MCB-1906060 and MCB-2145967) and the US National Institutes of Health (R15GM135893). Funders had no role in the study design, in the collection, analysis, and interpretation of the data, in the writing of the report, and in the decision to submit the article for publication.

## Conflict of Interest

The authors declare that the research was conducted in the absence of any commercial or financial relationships that could be construed as a potential conflict of interest.

## Publisher’s Note

All claims expressed in this article are solely those of the authors and do not necessarily represent those of their affiliated organizations, or those of the publisher, the editors and the reviewers. Any product that may be evaluated in this article, or claim that may be made by its manufacturer, is not guaranteed or endorsed by the publisher.

## Footnotes

^1^ http://rna.bgsu.edu/rna3dhub/motif/view/HL_27353.2
